# The role of the endosomal sorting complexes required for transport (ESCRT) in tumorigenesis

**DOI:** 10.3109/09687688.2014.894210

**Published:** 2014-03-18

**Authors:** Claudia Mattissek, David Teis

**Affiliations:** ^a^ Division of Cell Biology, Biocenter, Innsbruck Medical University InnsbruckAustria

**Keywords:** Endosomes, ESCRT, tumorigenesis

## Abstract

The endosomal sorting complexes required for transport (ESCRT) are needed for three distinct cellular functions in higher eukaryotes: (i) Multivesicular body formation for the degradation of transmembrane proteins in lysosomes, (ii) midbody abscission during cytokinesis and (iii) retroviral budding. Not surprisingly, loss of ESCRT function has severe consequences, which include the failure to down-regulate growth factor receptors leading to deregulated mitogenic signaling. While it is clear that the function of the ESCRT machinery is important for embryonic development, its role in cancer is more controversial. Various experimental approaches in different model organisms arrive at partially divergent conclusions regarding the contribution of ESCRTs to tumorigenesis. Therefore the aim of this review is to provide an overview on different model systems used to study the role of the ESCRT machinery in cancer development, to highlight common grounds and present certain controversies in the field.

## Potential links between the ESCRT machinery and tumorigenesis

The endosomal sorting complexes required for transport (ESCRT) were initially identified as genes required for vacuolar protein sorting (*vps*) in yeast (Bankaitis et al., 1986; Rothman & Stevens, 1986). The vacuole in yeast is functionally equivalent to the lysosome in higher eukaryotes. Subsequently, ESCRT mutants were found to form an exaggerated pre-vacuolar structure, named the “class E” compartment, which was comprised of flat membrane stacks containing accumulating membrane proteins that were no longer degraded in the vacuole (Raymond et al., 1992). The term ESCRT was coined when it became clear that three of the class E-*vps* genes assembled into one protein complex, ESCRT-I, and were required for the ubiquitin-dependent sorting of integral membrane proteins into the vacuole via the multivesicular body (MVB) pathway (Katzmann et al., 2001; Odorizzi et al., 1998). Soon, it turned out that the basic set-up of the ESCRT machinery is evolutionary well conserved across the eukaryotic lineage (Babst et al., 2000; Bishop & Woodman, 2001; Leung et al., 2008). One fundamental function of the ESCRT machinery, cargo sorting and intraluminal MVB vesicle (ILV) formation, can be studied in yeast without the additional complexity of multiple isoforms and possible redundancy in ESCRT machineries of metazoans.

In its most basic setting, the ESCRT machinery consists of five distinct protein complexes: ESCRT-0, ESCRT-I, ESCRT-II, ESCRT-III and the AAA-ATPase complex Vps4. They sequentially recruit one another from the cytoplasm onto the surface of the endosomal membrane to sort ubiquitinylated membrane proteins (cargo) from the limiting endosomal membrane into growing ILVs (reviewed in Henne et al., 2011; Schmidt & Teis, 2012). Ubiquitinylated membrane proteins arrive on endosomes either via endocytosis from the plasma membrane (endocytic cargo) or they originate from the trans-Golgi network (biosynthetic cargo). The MVB pathway is initiated by the recruitment of ESCRT-0 complex onto endosomes. ESCRT-0 binds via its FYVE domain to phosphatidylinositol-3-phosphate (PI3P). ESCRT-0 subsequently recruits ESCRT-I and ESCRT-II. ESCRT-0, -I and -II have distinct ubiquitin interaction motives to bind directly to ubiquitinylated membrane proteins. By doing so, these early ESCRT complexes recognize and gather cargo destined to be degraded via the MVB pathway in lysosomes. In the next step of the MVB pathway, the endosomal membrane is invaginated to generate an ILV that will ultimately bud into the endosomal lumen. ILV biogenesis requires the assembly of the ring-like ESCRT-III complex, which encircles cargo proteins and simultaneously drives membrane deformation and final scission. Prior to vesicle scission, cargo molecules are de-ubiquitinylated for ubiquitin recycling and reuse. Finally, Vps4 interacts with ESCRT-III and disassembles the ESCRT machinery back into the cytoplasm thereby terminating the MVB pathway (reviewed in Hanson & Cashikar, 2012; McCullough et al., 2013). When the limiting membrane of mature MVBs fuses with lysosomes, the cargo-laden ILVs are released into the acidic lumen of the lysosome where they are degraded by lysosomal hydrolases.

Besides their essential role in the MVB pathway, several ESCRT complexes also function in different processes such as viral budding and cytokinesis. First, the ESCRT-I protein Tsg101 was found to be required for retroviral budding (Garrus et al., 2001; Martin-Serrano et al., 2001; VerPlank et al., 2001). Enveloped viruses can hijack also other proteins from the ESCRT machinery to accomplish budding of their membrane envelope towards the extracellular space (Martin-Serrano et al., 2003; von Schwedler et al., 2003).

Evidence from plants (*Arabidopsis thaliana elch* mutants) (Spitzer et al., 2006) and mammalian cells called the attention to cytokinesis as another ESCRT-mediated process (Carlton & Martin-Serrano, 2007; Morita et al., 2007). Interestingly, ESCRT-III and Vps4 orthologues were also found in crenarchaea like *Sulfolobus acidocaldarius*, where a three-gene operon with shared common ancestry and similar mechanistic features to ESCRT proteins was shown to mediate cell division (Lindas et al., 2008; Samson et al., 2008).

The ESCRT machinery executes membrane budding and scission events away from the cytoplasm, which is topologically opposite to cellular membrane budding events that occur during endocytosis and secretion, where the membrane is pulled into the cytoplasm. This unusual ESCRT-mediated topology is the common feature in MVB biogenesis, the budding of enveloped viruses, and midbody abscission in cytokinesis. For cytokinesis and viral budding, ESCRT-0 and ESCRT-II and in some viruses also ESCRT-I appear to be dispensable, because ESCRT-III gets recruited to its target membranes by alternative adaptor proteins. For midbody abscission, centrosomal protein CEP55 recruits ESCRT-I protein Tsg101 and Alix (Agromayor & Martin-Serrano, 2013). Enveloped viruses use motifs in viral GAG proteins, called late assembly domains, to bind Tsg101 or other ESCRT associated proteins such as Alix, Nedd4 or Itch (Votteler & Sundquist, 2013). Hence, ESCRT-III subunits and Vps4 are required for all known ESCRT-mediated processes.

## ESCRT-mediated lysosomal degradation of activated EGFR

Growth factor receptors, but also adhesion molecules like integrins, adherens- and tight junction proteins are clients of the ESCRT machinery as they are degraded via the MVB pathway in lysosomes (Babst, 2005; Lobert et al., 2010; Tu et al., 2010). Alterations in the abundance of those transmembrane proteins could modify the tumorigenic potential of cells. One such scenario is the impact of ESCRT malfunction on epithelial cell polarity, which is based on the proper localization and recycling of tight- and adherens junction proteins and the associated polarity complexes. Loss of epithelial polarity can result in epithelial to mesenchymal transition (EMT). During EMT, polarized epithelial cells convert into a migratory fibroblastoid state with increased cell motility, which is suspected to facilitate metastasis (Boyer et al., 1993; Dukes et al., 2011; Gotzmann et al., 2004).

Most frequently, the effects of ESCRT depletion on transmembrane protein degradation and cell signaling are studied using the epidermal growth factor receptor (EGFR) as a model growth factor receptor. How loss of ESCRT function and the subsequent block in EGFR degradation affects downstream signaling, appears to be surprisingly difficult to predict. Early on, mouse fibroblast cells with a mutation causing low expression of the ESCRT-I protein Tsg101 were reported to rapidly recycle EGFR back to the cell surface. As a result, they had increased receptor signaling and higher activity of mitogen-activated protein kinase (MAPK) signaling reflected by prolonged ERK1/2 phosphorylation (Babst et al., 2000). Regarding the impact on signaling downstream of EGFR, it seems to matter at which step of the MVB pathway the degradation of EGFR is blocked. Knockdown of Hrs (ESCRT-0), Tsg101 (ESCRT-I), Eap30 (ESCRT-II) as well as Chmp3 (ESCRT-III) impaired EGFR degradation (Bache et al., 2006) and overexpression of Hrs (ESCRT-0) reduced EGF mediated Stat3 activation (Scoles et al., 2005), accordingly. Only the knockdown of ESCRT subunits 0 (Hrs) and I (Tsg101 and Vps37) caused continuous EGFR activation and MAPK signaling (Raiborg et al., 2008). Consistently, over-expression of mutant Hrs that can no longer bind to ubiquitinylated membrane proteins resulted in delay of EGFR degradation (Urbe et al., 2003). However, continued MAPK signaling was not seen for EAP30 (ESCRT-II) or Chmp3 (ESCRT-III) depletion. Sustained MAPK signaling could be caused by enhanced recycling of endocytosed EGFRs upon depletion of Hrs (ESCRT-0) or Tsg101 (ESCRT-I), which was not observed when Eap30 (Vps22, ESCRT-II) or Chmp3 (Vps24, ESCRT-III) were depleted (Raiborg et al., 2008).

Similar to Tsg101, Vps37A (ESCRT-I) knockdown caused prolonged EGFR activation as well as hyper-activation of downstream AKT and MAPK signaling (Wittinger et al., 2011). Thus, termination of EGFR signaling may occur prior to ESCRT-II engagement (Malerod et al., 2007), just before the EGFR will enter an ILV. The body of evidence suggesting that termination of EGFR signaling occurs prior to ESCRT-II engagement is challenged by other publications: A reduction of Tsg101 (ESCRT-I) compromised the MAPK/ERK signaling pathway (Zhang et al., 2011) and Tsg101 deletion down-regulated EGFR protein levels post-transcriptionally (Morris et al., 2012).

In *Drosophila*, not only ESCRT-0 and ESCRT-I but also ESCRT-II and -III complexes were required to prevent excess receptor signaling (Vaccari et al., 2009). If the AAA-ATPase Vps4B was down-regulated by *sh*RNA or a dominant negative Vps4 mutant was expressed, EGFR was hyper-activated and accumulated within the cells (Lin et al., 2012). On the other hand, Vps4A, as well as Chmp6, has been shown to be essential for Ras-induced cellular transformation and recycling of Ras and the EGFR back to the plasma membrane (Zheng et al., 2012). Tumorigenic Ras mutations and Vps4 up-regulation could have a synergistic effect on cellular transformation.

Overall, the majority of publications provide clear evidence for ESCRT-dependent EGFR degradation via MVBs in lysosomes. In many cases, impaired EGFR degradation appears to hyper-activate downstream signaling. Surprisingly, the accumulation of activated EGFR on endosomes seems to have little effect on transcriptional programs. Interfering with receptor endocytosis on the other hand and thereby keeping the activated EGFR at the plasma membrane affected EGF-induced transcripts, comparable to EGFR overexpression (Brankatschk et al., 2012).

**Table 1.** Summary.

Model system → ESCRT protein ↓	*Drosophila*	Mammals *in vitro/in vivo*	Tumor samples
Hrs (ESCRT-0) (Vps27)	***TS****(Lloyd et al.,*^2002^; *Woodfield et al.,*^2013^*)*	**O** (Scoles et al., 2005; Toyoshima et al., 2007)	**up** (Toyoshima et al., 2007)
Tsg101 (ESCRT-I) (Vps23)	***TS****(Moberg et al.,*^2005^*; Thompson et al.,*^2005^*; Vaccari et al.,*^2009^*)*	**O** (Carstens et al., 2004; Krempler et al., 2002; Liu et al., 2010; Morris et al., 2012; Oh et al., 2007; Ruland et al., 2001; Wagner et al., 2003; Young et al., 2007a; Zhang et al., 2011; Zhu et al., 2004) ***TS****(Dukes et al.,*^2011^*; Li & Cohen,*^1996^*; Oh et al.,*^2007^*; Young et al.,*2007b*)*	**noRole** (Carney et al., 1998; Steiner et al., 1997) **up** (Liu et al., 2002; Liu et al., 2010; Oh et al., 2007; Toyoshima et al., 2007) ***down****(Cai et al.,*^2008^*; Lu et al.,*^2007^*)*
Vps37A (ESCRT-I) (Vps37)		***TS****(Wittinger et al.,*^2011^*; Xu et al.,*^2003^*)*	***down****(Wittinger et al.,*^2011^*; Xu et al.,*^2003^*)*
UBAP1 (ESCRT-I) (UBAP1)			***down****(Xiao et al.,*^2006^*)*
EAP45 (ESCRT-II) (Vps36)	***TS****(Vaccari et al.,*^2009^*; Woodfield et al.,*^2013^*)*		
EAP30 (ESCRT-II) (Vps22)			
EAP20 (ESCRT-II) (Vps25)	***TS****(Herz et al.,*^2006^*; Thompson et al.,*^2005^*; Vaccari & Bilder,*^2005^*; Vaccari et al.,*^2009^*; Woodfield et al.,*^2013^*)*		
Chmp3 (ESCRT-III) (Vps24)		**O** (Dukes et al., 2011; Wilson et al., 2001; Walker et al., 2006)	**up** (Walker et al., 2006)
Chmp1A (ESCRT-III) (DID2)		***TS****(Li et al.,*^2008^*)*	***down****(Li et al.,*^2008^*)*
Chmp4C (ESCRT-III) (Snf7)			**up** (Nikolova et al., 2009) **SNPs** (Pharoah et al., 2013)
Vps4B (Vps4)		***TS****(Lin et al.,*^2012^*; Liao et al.,*^2013^*)*	***down****(Lin et al.,*^2012^*)*

**Oncogenic potential (O)**: pro-proliferative/loss is anti-proliferative/OE is pro-proliferative/**up**-regulated in tumors. ***Tumor suppressive potential* (*TS*)**: anti-proliferative/loss is pro-proliferative/OE is anti-proliferative/**down**-regulated in tumors.

In conclusion, the only common feature observed in all model systems used, is the failure to degrade signaling receptors, either accompanied by enhanced receptor recycling or accumulation of activated receptors on endosomes. Nevertheless, this does not always seem to directly translate into transforming properties of cells. Therefore we will subsequently discuss in detail how the ESCRT machinery may contribute to tumor development. A summary is provided in Table 1.

## ESCRTs in unicellular organisms

Structure and function of ESCRT proteins are evolutionary well conserved from unicellular organisms to mammals. Are there lessons to be learned from unicellular organisms on proliferation properties of ESCRT mutants? The evolutionary oldest organism with ESCRT proteins, the crenarchaeon *Sulfolobus acidocaldarius* requires orthologs of the ESCRT machinery for cell division (Lindas et al., 2008; Samson et al., 2008). In yeast, ESCRTs function is not required for cell division and restricted to the MVB pathway. Loss of any ESCRT protein in yeast leads to a block in transmembrane protein degradation and class E compartment formation. Under optimal conditions, loss of ESCRT function does not affect growth, but recent work has provided first evidence that growth of ESCRT-deficient yeast is sensitive to nutritional or environmental stress (Jones et al., 2012). This might reflect deficits in responding to extracellular cues maybe somewhat reminiscent of the defects caused by impaired transmembrane protein turnover in higher organisms.

Overall, ESCRT-depletion in unicellular organisms has shown to be anti-proliferative. These findings provoke important questions about the response of single cells and whole tissues to the combination of membrane protein accumulation, like growth factor receptors, and the proliferation disadvantage due to lack of ESCRT function.

## ESCRTs in *Drosophila*

Perhaps the strongest evidence for a role of the ESCRT machinery in tumor suppression is provided by genetic studies using *Drosophila melanogaster* as a model system.

* ESCRT-0*: Hrs (ESCRT-0) loss-of-function mutants suffer from early pupal lethality. Cells lacking functional Hrs fail to degrade active EGF and *Torso* tyrosine kinase receptor, which leads to enhanced signaling and defects in embryonic patterning. These data suggest that Hrs and MVB formation function to down-regulate receptor tyrosine kinase signaling (Lloyd et al., 2002).
 *ESCRT-I*: *Drosophila* genetics enable engineering of mosaic tissues, suitable to investigate the interaction among mutant and wild-type cells. Patches of tissue that are deficient for the ESCRT-I subunit Tsg101 accumulate Notch on endosomes. This causes ectopic expression of the Notch target gene *unpaired*, a secreted ligand activating the JAK-Stat pathway in surrounding wild-type cells, correlated with non-cell autonomous hyperproliferation of the neighboring wild-type cells (Moberg et al., 2005).
 *ESCRT-II*: For the ESCRT-II component Eap20 (Vps25), a similar scenario with non-cell autonomous neoplastic transformation of wild-type cells surrounding ESCRT mutants due to continuous Notch signaling could be shown. As for Tsg101 (ESCRT-I), increased Notch activity led to production of the mitogenic JAK-Stat pathway ligand *unpaired* (Vaccari & Bilder, 2005). Interestingly, this loss of ESCRT function is accompanied by apoptosis of the mutant cells (Herz et al., 2006). Furthermore, the apoptosis in Eap20 mutant cells has been tracked down to their sensitivity to cell competition: When apoptosis is blocked, the ESCRT mutants no longer die but begin to hyper-proliferate as well. Interestingly, Hrs (ESCRT-0) knockdown unlike Tsg101 (ESCRT-I) and Eap20 (ESCRT-II) knockdown, did not activate Notch signaling in a cell culture assay measuring Notch activation through luciferase activity (Thompson et al., 2005). To study the cell-autonomous phenotype of ESCRT-II loss-of-function-mutants, tissues predominantly mutant for ESCRT-II components Eap45 (Vps36), Eap30 (Vps22) and Eap20 (Vps25) were generated. They display many characteristics of neoplastic transformation; The ESCRT-II mutants up-regulate Notch, JAK-Stat and JNK signaling but are again prone to apoptosis (Woodfield et al., 2013).

These findings suggest that ESCRT mutations alone may not be sufficient for tumorigenesis but could accelerate the growth of cells no longer able to undergo apoptosis. Similarly, mutations in other key regulators of the endocytic pathway like the syntaxin *avalanche* or the Rab-GTPase Rab5 (Lu & Bilder, 2005) increased the tumorigenic potential, indicating that endocytosis may generally function as a tumor suppressor pathway (Mosesson et al., 2008).

A comparative analysis of ESCRT-I, -II and -III also came to the conclusion that the ESCRT machinery is required to prevent excess Notch and EGFR signaling. The same study proposed a slightly different role of at least some of the ESCRT-III components [Vps20 (Chmp6), Vps32 (Snf7) and Vps2 (Chmp2)] in *Drosophila* as the respective mutants displayed reduced degrees of cell proliferation compared to ESCRT-I and ESCRT-II mutants. Moreover, MVB biogenesis still persisted to some degree in ESCRT-III mutant cells. As their screen did not pick up the ESCRT-0 proteins Hrs and Stam, the authors propose ESCRT-I to be able to substitute for ESCRT-0 in *Drosophila* (Vaccari et al., 2009).

Taken together, loss of ESCRT function in *Drosophila* can promote neoplastic transformation but also lead to loss of cell polarity and render the cells prone to apoptosis. Sustained growth factor signaling boosts non-cell autonomous over-proliferation in the adjacent wild-type cells. Only when cell death is blocked in the ESCRT mutants, they overgrow wild-type tissue. Therefore it seems that the MVB pathway is required to balance cell survival and proliferation in developing tissues.

## Zebrafish

In the zebrafish (*Danio rerio*), a vertebrate model system, knockdown of ESCRT subunits led to severe developmental defects, like in other multicellular organisms:

 *ESCRT-I*: Knockdown of ESCRT-I subunit Vps37A with morpholinos yielded a phenotype with significantly reduced mobility (Zivony-Elboum et al., 2012).
 *ESCRT-III*: A knockdown of Chmp1a, an ESCRT-III associated protein, had devastating effects on brain development (Mochida et al., 2012). To our knowledge, the interplay of ESCRT malfunction and tumorigenesis in zebrafish has not yet been investigated.

## Mammals: Mouse models and *in vitro* experiments

The requirements for ESCRT proteins during development have been excellently reviewed in (Rusten et al., 2012). All available ESCRT-deficient mice – Hrs, Stam1/2, Tsg101 and Chmp5 – display severe developmental defects and early embryonic lethality.

 *ESCRT-0*: Hrs (ESCRT-0) null embryos developed with their ventral region outside of the yolk sac, had two independent bilateral heart tubes (*cardia bifida*), lacked a foregut and died around embryonic day E11. Enlarged early endosomes were detected in the mutant cells of several tissues including definitive endoderm, suggesting that a deficiency in vesicular transport via early endosomes underlies the mutant phenotype (Komada & Soriano, 1999). Similarly, Stam1/2 double knockout mice die during embryonic development at around embryonic day E11 (Yamada et al., 2002). Cell culture data for Hrs support an important role in normal cellular homeostasis. Hrs overexpression reduced EGFR abundance and EGF-mediated Stat3 activation in rat schwannoma cells (Scoles et al., 2005). Hence, Hrs overexpression reduced the presence of total and active EGFR. Down-regulation of Hrs by *si*RNA reduced the tumorigenic potential by inhibiting cell colony formation and metastasis of HeLa cells due to increased E-Cadherin expression (Toyoshima et al., 2007).
 *ESCRT-I*: Homozygous Tsg101-/- (ESCRT-I) embryos displayed embryonic lethality and failed to develop past day E6.5. The mutant embryos were reduced in size and did not form a mesoderm. Tsg101 was essential for the proliferative burst before the onset of gastrulation and its knockout caused an accumulation of the tumor suppressor p53 (Brown et al., 2009). The accumulation of p53 was most likely caused by a regulatory feedback loop between Tsg101 and the E3-Ubquitin Ligase MDM2. Tsg101 was found to inhibit Mdm2’s self-mediated decay and thus down-regulating p53 levels (Li et al., 2001). Notably, the reduced size of Tsg101-/- knockout embryos was, other than in Tsg101 *Drosophila* mutants, not associated with increased apoptosis (Ruland et al., 2001). These *in vivo* findings are difficult to align with *in vitro* tissue culture experiments where Tsg101 was down-regulated on *m*RNA level: Already before the ESCRT machinery as a whole was discovered, depletion of ESCRT-I subunit Tsg101 by anti-sense RNA was associated with NIH 3T3 fibroblast transformation and hence the gene termed *tumor susceptibility gene 101*. Knockdown of Tsg101 in monolayer cultures induced cellular transformations such as focus formation and anchorage independent growth in soft agar. Cell clones lacking Tsg101 were able to give rise to metastatic tumors in nude mice (Li & Cohen, 1996). Thereafter, ESCRT proteins were generally regarded as tumor suppressor genes. The same group published evidence for the Tsg101 gene to be mutated at high frequency in human breast cancers (see below), but retracted the conclusions soon after (Li et al., 1998). Nevertheless, the association between Tsg101 and tumorigenesis made Tsg101 the best-researched ESCRT protein with regard to neoplasia. Tsg101 knockdown in monolayers of epithelial cells caused loss of their polarized organization, interfered with the establishment of a normal epithelial permeability barrier and reduced the formation of correctly polarized three-dimensional cysts. These defects were due to impaired recycling of tight junction protein Claudin-1 and its subsequent intracellular accumulation (Dukes et al., 2011).

However, Tsg101 was also found to support proliferation: Deletion of Tsg101 in mammary epithelia (using MMTV-Cre) did not result in breast cancer development in mice but impaired proliferation of the Tsg101 deficient cells *in vitro* (Wagner et al., 2003). It is not clear if these proliferation deficits are caused by defects in cytokinesis that required Tsg101. Likewise, Tsg101 was shown to be essential for cell proliferation and cell survival also in other cells (Krempler et al., 2002; Zhu et al., 2004). Moreover, Tsg101 ablation in mouse tissue led to cell cycle arrest at G_1_/S and p53 independent cell death (Krempler et al., 2002), evocative of *Drosophila* tissues, where ESCRT mutant cells are more prone to cell death. To which degree the cell cycle arrest and cell death are sensed and mediated by the tumor suppressor p53 remains controversial (Carstens et al., 2004).

The pro-proliferative role of Tsg101 itself appears to extend to tumor cells as a reduction of Tsg101 protein by *si*RNA had a negative impact on tumor cell growth (Zhu et al., 2004) and compromised the MAPK/ERK signal pathway in breast cancer cells (Zhang et al., 2011). Moreover, Tsg101 does promote growth in NIH3T3 fibroblasts as well as in lung cancer cells (Liu et al., 2010). One study, using conditional Cre-mediated deletion of Tsg101 in mouse embryonic fibroblasts, reports the post-transcriptional down-regulation of the EGFR upon Tsg101 depletion and induction of autophagy prior to cell death (Morris et al., 2012). In an experimental set-up, where Tsg101 was overexpressed in the developing mammary gland, a weak oncogenic potential could be noted with Tsg101-overexpression resulting in increased signaling through ERK1/2 and Stat3/5 and development of mammary gland abnormalities and mammary cancer after a long latency (Oh et al., 2007). In support of these findings, human ovary SKOV-3 cells transfected with Tsg101 *si*RNA formed smaller tumors in athymic nude mice (Young et al., 2007b). In addition to its essential role in the MVB pathway, the role of Tsg101 in cytokinesis has also been associated with the tumor suppressor gene BRCA2. BRCA2 is a major breast cancer susceptibility gene and is involved in the repair of double-strand breaks (Foulkes & Shuen, 2013). Inactivation or depletion with *si*RNA of BRCA2 increases cytokinetic failures (Daniels et al., 2004). A recent publication claims that BRCA2 is a component of the midbody, the electron dense structure separating daughter cells during cell division, where it interacts with the CEP55 and the ESCRT components Alix and TSG101. BRCA2 missense mutations lead to CEP55, Alix and Tsg101 mislocalization and subsequently drive multinucleation and unresolved cytokinetic bridges (Mondal et al., 2012). However, this finding is controversial, as it had been shown earlier, that tumor suppressor protein BRCA2 does not regulate cytokinesis in human cells (Lekomtsev et al., 2010).

As for Tsg101, there is some evidence for a potential tumor suppressor role of another ESCRT-I subunit, Vps37A. Vps37 has four isoforms (Vps37A-D). Each of them can be a component of ESCRT-I together with Tsg101, Vps28 and hMVB12A/B. Overexpression of Vps37A inhibited both anchorage-dependent and -independent cell growth *in vitro*. Consistently, *sh*RNA knockdown resulted in enhanced cell growth and invasive ability of the cells (Xu et al., 2003). In an inducible human ovarian cancer cell culture model, knockdown of Vps37A was found to cause cytoplasmic pEGFR retention and hyper-activation of downstream AKT and MAPK signaling. This resulted in invasive growth into a collagen matrix. In addition, xenografts displayed enhanced growth in nude mice and increased invasion of a collagen matrix (Wittinger et al., 2011).

 *ESCRT-III*: The ESCRT-III subunit Chmp3 appears to be less directly linked to cancer. Overexpression of Chmp3 in prostate cancer cells induced neuroendocrine differentiation and an increase in neuroendocrine cell numbers (Wilson et al., 2001), which correlates with progressive cancer, and poor prognosis in many tumor types. Consistently, neuroendocrine-like differentiation in the poorly differentiated non-small cell lung carcinoma cells is associated with the up-regulation of Chmp3 (Walker et al., 2006). Expressing a dominant negative form of Chmp3 in MDCK cells caused recycling defects of the tight junction protein Claudin-1 and its accumulation in intracellular vesicles (Dukes et al., 2011).

Reduction of another ESCRT-III subunit, Chmp1A, by *sh*RNA enhanced the tumorigenic potential by increasing anchorage-independent growth in HEK 293T cells and led to tumor formation in a xenograft assay in athymic mice. Conversely, Chmp1A overexpression was accompanied by p53 accumulation and inhibited tumor xenograft growth of human pancreas carcinoma PanC-1 cells. This is indicative of Chmp1A as a tumor suppressor protein in pancreas (Li et al., 2008).

 *Vps4-complex*: Vps4B has been linked to hypoxia and cellular transformation in a human breast cancer cell model (Lin et al., 2012). Hypoxic conditions, frequently found in the center of larger tumor entities, induced a down-regulation of Vps4B by the ubiquitin proteasome system. Depletion by *sh*RNA or expression of a dominant negative Vps4 mutant blocked EGFR degradation and enhanced EGFR signaling, which in turn promoted anchorage-independent growth and resistance to *Gefitinib*, a tyrosine kinase inhibitor mainly affecting EGFR kinases (Lin et al., 2012). Interestingly, the glycolytic pathway is down-regulated in VPS4B-depleted human mammary gland SKBR3 cells, suggesting a potential crosstalk between the abnormal metabolism of cancer cells and MVB dysfunction (Liao et al., 2013). Overall, it seems that neither *in vivo* model organisms nor *in vitro* cell culture experiments offer a simple or unifying answer to the role of the ESCRT machinery in tumorigenesis.

## Tumor samples

A rather small set of studies on human cancer tissues provides evidence for a deregulation of ESCRT function in certain human cancers:

 *ESCRT-0*: The ESCRT-0 protein Hrs was found to be up-regulated in tumor specimens of different origin (stomach, colon, liver, cervix and melanoma) (Toyoshima et al., 2007).
 *ESCRT-I*: Loss-of-function mutations in Tsg101 were initially described to be involved in human breast carcinomas (Li et al., 1997). However, further studies could find some deletions or aberrant splicing but no evidence suggesting that loss of this putative tumor suppressor gene plays a role in the molecular pathogenesis of the cancers (Carney et al., 1998; Steiner et al., 1997); this discrepancy led to retraction of the original publication (Li et al., 1998). Tsg101 might not be a tumor suppressor gene as initially predicted, but it could nevertheless play a role in cancer, as its up-regulation has been associated with a poor prognosis in ovarian cancers (Young et al., 2007a). Further evidence describes Tsg101 up-regulation in tumor malignancies like breast cancer (Oh et al., 2007), papillary thyroid (Liu et al., 2002) and colorectal carcinomas (Ma et al., 2008). Regarding the expression levels of Tsg101 in lung cancer, different results have been reported. In one study, Tsg101 was found to be up-regulated in 15 lung cancer cell lines and five lung cancer tissue specimens (Liu et al., 2010). Other studies described Tsg101 levels to be decreased in different human lung cancer samples (Cai et al., 2008; Lu et al., 2007); reviewed in (Jiang et al., 2013).

As far as it has been investigated, the picture is less ambiguous for another ESCRT-I subunit, Vps37A: Vps37A *m*RNA levels were lowered and protein level was significantly reduced in the majority hepatocellular carcinomas analyzed. Therefore Vps37A was suggested to be a growth inhibitory protein required to decrease the invasion of hepatocellular carcinoma cells (Xu et al., 2003). Beyond that, Vps37A *m*RNA was significantly down-regulated in ovarian cancer samples. This seemed to modify the prognostic value of EGFR and HER2 expression, which were hyper-activated as mentioned above, and had clinical relevance leading to resistance to *Cetuximab*, a therapeutic EGFR antibody (Wittinger et al., 2011).

As part of an endosome-specific ESCRT-I complex, UBAP1 is required for the degradation of EGFR (Stefani et al., 2011). UBAP1 had initially been linked to a region with common loss of heterozygosity in nasopharyngeal carcinomas (Qian et al., 2001). In a tissue microarray from nasopharyngeal carcinomas, down-regulation on protein level was found (Xiao et al., 2006).

 *ESCRT-III*: The *m*RNA of the ESCRT-III subunit Chmp1A was deregulated in many tumor types including skin tumors and strongly and consistently down-regulated in a number of pancreatic tumors judging from a screen of human cancer arrays and pancreatic tissue arrays. Its protein expression was mostly reduced in various pancreatic ductal adenocarcinomas compared with normal ducts (Li et al., 2008). Another ESCRT-III subunit, Chmp4C, was frequently overexpressed in ovarian carcinoma tissue, and not expressed in control tissues (Nikolova et al., 2009). Genome wide association studies identified Chmp4C as a candidate susceptibility gene contributing to an excess familial risk to epithelial ovarian cancers (EOC) and found it to be overexpressed in two epithelial ovarian cancer cell lines and in primary EOC tissues (Pharoah et al., 2013). The additional risk may be due to Chmp4C’s function in the Aurora B-mediated abscission checkpoint, NoCut, that controls the timing for the resolution of intercellular chromatin bridges (Carlton et al., 2012).
 *Vps4-complex*: Also in breast tumors, a connection between down-regulation of Vps4B and tumor malignancy has been made. Vps4B *m*RNA levels were down-regulated in high grade or recurrent breast tumors (Stage IV) in comparison to lower grade tumors (Stage II–III). Moreover, low Vps4B *m*RNA levels correlated with high EGFR protein levels and high Vps4B *m*RNA levels with low EGFR abundance (Lin et al., 2012).

Overall, Hrs (ESCRT-0) and Tsg101 (ESCRT-I) seem to be mostly up- and Vps37A (ESCRT-I), Chmp1A and Vps4B mostly down-regulated in tumor samples. This pattern is frequently reflected by *in vitro* cell culture experiments (discussed above) from the same studies.

## Concluding remarks

There are examples for certain tumor entities showing preferentially up- or down-regulation of ESCRT components suggesting that tumors cells indeed benefit from alterations in the ESCRT machinery. *Drosophila* experiments indicate that ESCRT mutations generally tend to render cells prone to apoptosis, particularly in competition with wild-type cells. It is therefore tempting to speculate that loss of ESCRT function, although initially not beneficial for the cell, could become advantageous in a subset of tumor cells that have already accumulated anti-apoptotic mutations. An up-regulation of ESCRT proteins on the other hand could speed up transmembrane protein turnover and thereby ease the adaption to changes in the cellular environment.

The role of ESCRTs in tumorigenesis is generally attributed to defective transmembrane protein homeostasis and the resulting consequences on cell signaling. Nevertheless, it is also conceivable that tumorigenic potential could be accounted for by cytokinetic defects that result in polyploidy or even a synergistic effect of both, defects in the MVB pathway and cytokinesis. Potential mechanisms and examples for cell cycle and cytokinesis defects in human cancers have been recently reviewed (Sagona & Stenmark, 2010). Furthermore, the ESCRT machinery is implied in autophagy and exosome generation. As exosomes contribute to intercellular communication, it is just as well feasible, that this ability of the ESCRT machinery could affect tumorigenicity, too (Théry, 2011).

While more and more potential links between the ESCRT machinery and cancer are identified, a unifying concept of how the ESCRT machinery or loss thereof would contribute to cancer is not yet emerging. Given that these proteins should function together as part of one large multi-subunit machinery, it remains unclear how different components of the same machinery would affect cell growth in different ways.

## Declaration of interest

The authors report no conflicts of interest. The authors alone are responsible for the content and writing of the paper.

Funding was provided by HFSP-CDA-00001/2010-C, Austrian Science Fund (FWF), Y444-B12 and SFB021 (F21) to DT.
